# Systemic Lupus Erythematosus Presenting as Myopericarditis with Acute Heart Failure: A Case Report and Literature Review

**DOI:** 10.1155/2019/6173276

**Published:** 2019-07-24

**Authors:** Richard Jesse Durrance, Malahat Movahedian, Worku Haile, Katerina Teller, Richard Pinsker

**Affiliations:** ^1^Department of Medicine, Jamaica Hospital Medical Center, 8900 Van Wyck Expressway, Jamaica, NY 11418, USA; ^2^Department of Rheumatology, Jamaica Hospital Medical Center, 8900 Van Wyck Expressway, Jamaica, NY 11418, USA

## Abstract

Acutely decompensated dilated cardiomyopathy in a middle-aged patient without the typical risk factor profile presents a clinical dilemma. While cardiomyopathy is a known aspect of systemic lupus erythematosus (SLE), initial clinical presentation as decompensated dilated cardiomyopathy (DCM) is exceedingly rare in the literature. We share the case of a 49-year-old African-American female with no past medical history who presented with overt heart failure of 4 weeks evolution. Workup showed acute onset decompensated dilated cardiomyopathy, with a serologic profile compatible with SLE. She responded well to immunosuppressive steroid therapy. Literature review for SLE presenting as dilated cardiomyopathy with acute heart failure revealed a paucity of clinical evidence and consensus. Therefore, a comprehensive review of case reports was undertaken. A total of 10 cases were identified. Patients were 90% female and averaged 31 years of age. Dyspnea was the most common clinical presentation, and dilated cardiomyopathy with severely compromised left ventricular function was universally appreciated. Clinical presentation to diagnosis averaged 2 weeks. Immunosuppressive therapy regimens were universally employed; however, the regimens varied significantly. High-dose steroid therapy was most commonly used, and clinical and functional recovery was reported in 90% of individual case reports. Within the limited evidence and experience of therapeutic approaches, the efficacy of different singular or combined therapy is based solely on anecdotal case reports. Given the near-complete response to a short course of high-dose steroid therapy as much in the clinical recovery as in the resolution of DCM, the limited evidence based on review of these observational case studies and series supports the initial use of high-dose steroid therapy in acute lupus myocarditis.

## 1. Introduction

Acute heart failure, defined as the rapid change in signs and symptoms of right or left ventricular overload requiring urgent medical management, accounts for more than one million hospitalizations per year [1]. While the vast majority are secondary to known chronic heart failure or coronary artery disease, the patient that defies both these categories provides a diagnostic challenge, which carries significant therapeutic and prognostic consequences.

Systemic lupus erythematosus (SLE), a multi-system autoimmune disorder affecting an estimated 300,000 people in the United States [1], typically presents with compromise of joints, skin, or the kidneys [2]. The cardiovascular implications of the disease have been well described, with both specific cardiac tissue involvement and a predisposition to atherosclerotic disease [3, 4]. Cardiac involvement has been reported to be present in greater than 50% of patients [5–7] and can affect any of the three layers of the cardiac tissue: endocardium, myocardium, or epicardium, as well as the pericardial serosa. However, while prevalence of cardiac involvement is elevated, the prevalence of dilated cardiomyopathy in SLE is not known. In addition, while postmortem studies have found prevalence of myocarditis greater than 50%, clinically significant myocarditis has been reported in approximately 9% of patients [7].

The autoantibody profile of cardiac involvement has been postulated, with suggested specificity for the cardiac tissue described: endocardial and valvular pathology has demonstrated association with the presence of anti-phospholipid and anti-cardiolipin antibodies [8], whereas myocarditis has been associated with anti-Ro antibodies; pericarditis, while seldom symptomatic, has not shown a particular autoantibody profile [5].

Given that the identification of SLE can be elusive and is often significantly delayed [9], and the etiologic diagnosis of heart failure is an important determinant in therapeutic management, the combination of SLE presenting with acute heart failure due to dilated cardiomyopathy presents a unique and only anecdotally recognized occurrence in the literature to date. However, while limited case reports and case series at tertiary care centers exist, a comprehensive compilation and review of these anecdotal cases has not, to our knowledge, been done. Therefore, we present the case of a middle-aged woman with no significant past medical history and no dermatologic, articular, or renal compromise, who developed overt systolic heart failure and cardiomegaly as the initial feature of SLE. A systematic literature review of SLE presenting as decompensated heart failure is also presented.

## 2. Case Presentation

**Figure 1:**
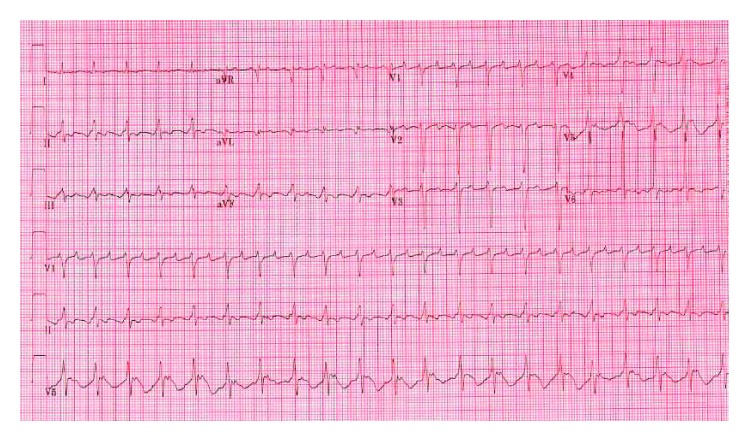
ECG of the patient on admission showing atrial flutter with a ventricular rate of 126 beats/minute and T wave inversions in inferior and lateral leads.

**Figure 2:**
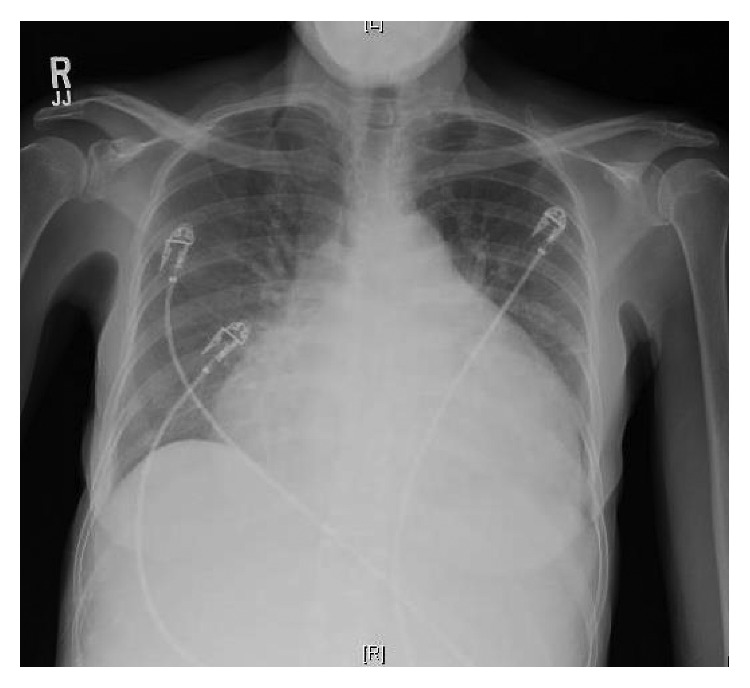
Chest X-ray of the patient on admission demonstrating markedly enlarged cardiac silhouette with “water-bottle” appearance.

**Figure 3:**
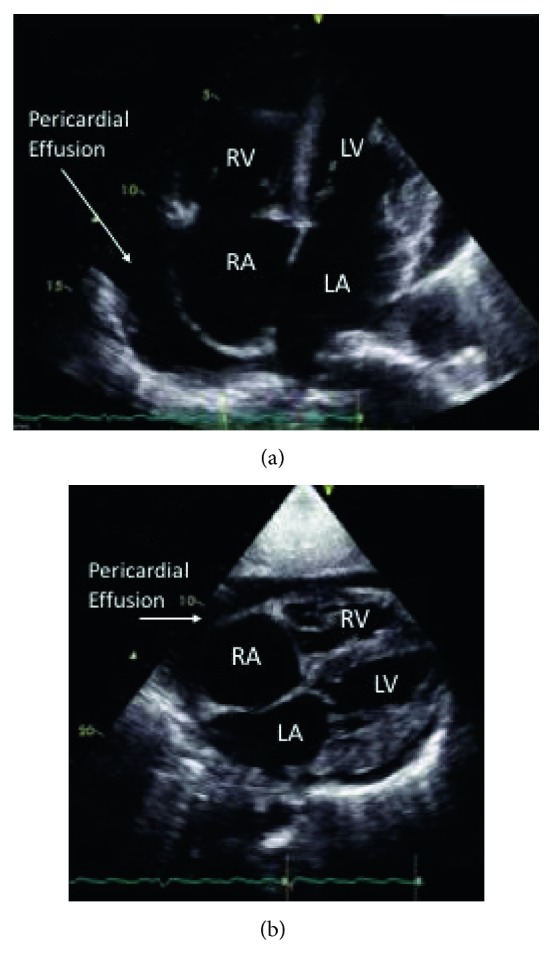
Transthoracic ultrasound on admission: (a) apical four-chamber view and (b) subcostal four-chamber view showing a large pericardial effusion and dilated cavities. On Doppler and dynamic views, global hypokinesis and severe mitral regurgitation were appreciated (RA = right atrium; RV = right ventricle; LA = left atrium; LV = left ventricle).

A 49-year-old Jamaican female with insignificant past medical history presented to the Emergency Department of an urban hospital with a chief complaint of progressively worsening shortness of breath of approximately 4 weeks evolution. Physical exam was remarkable for marked jugular venous distension, bilateral basal wet crackles, a distended and globally tender abdomen with positive fluid shift and positive hepatojugular reflex, as well as bilateral +2 lower leg edema. The patient's labs are shown in Table 1 and were remarkable for anemia, elevated troponin, markedly elevated pro-BNP, transaminases, mildly elevated TSH, and CRP. ECG tracing showed atrial flutter with a ventricular rate of 126 bpm (Figure 1). Chest X-ray showed a markedly enlarged cardiac silhouette with a water-bottle appearance suspicious for pericardial effusion (Figure 2), for which an echocardiogram was done on hospital day 1, and confirmed the presence of a significantly dilated right ventricle, severe tricuspid, and moderate mitral regurgitation with no valvular structural abnormalities described. In addition, a moderately compromised left ventricular ejection fraction (LVEF) (30–40%) and a large pericardial effusion (Figure 3) were shown. CT of the chest with pulmonary artery protocol was not supportive of pulmonary embolism as the underlying etiology. While the syndromic diagnosis of dilated cardiomyopathy with right sided heart failure was made, the etiology remained unknown.

The patient was started on beta-blocker and diuretic therapy, and with close monitoring began to improve from her heart failure. While aggressive medical management continued, an etiologic search for the underlying pathology began. Toxicology and infectious disease markers were negative. A viral panel was sent out, and later returned as unremarkable. A search for underlying malignancy also was negative. Left heart catheterization showed normal coronaries but was remarkable for severe mitral and tricuspid regurgitation. Right heart catheterization was performed due to the patient's persistently labile hemodynamics intermittently requiring pressor support suggestive of cardiogenic shock due to right heart failure and to establish an initial baseline of pulmonary artery pressure in newly diagnosed heart failure without clear etiology. This revealed severe mitral and tricuspid regurgitation with moderate pulmonary regurgitation and only mildly increased pulmonary artery pressure, thereby supporting the mechanism of decompensated heart failure as secondary to dilated cardiomyopathy.

It was only when ANA titers came back strongly positive with low C3 and C4 complement levels that the etiology was suspected to be most likely autoimmune. The patient was immediately started on prednisone (40 mg daily), and further workup showed elevated levels of both anti-double-stranded DNA and anti-Smith antibodies along with decreased levels of C3 and C4, confirming a diagnosis of SLE and lupus myopericarditis. On further examination of the patient, no other clinical findings of Lupus could be identified besides microcytic-hypochromic anemia.

On subsequent follow-up, the patient showed continuous improvement with steroid therapy and was ultimately scheduled for valve replacement surgery. As heart function monitoring and prednisone therapy continued, surgical intervention was ultimately averted. The patient progressed with complete functional recovery. A year later, EKG showed a normal sinus rhythm; ECHO analysis showed a LVEF of 55%; and the patient remained clinically stable on maintenance hydroxychloroquine and prednisone therapy, avoiding surgical intervention.

## 3. Literature Review

A comprehensive literature review of case reports and case series in which dilated cardiomyopathy was associated with the presentation of SLE was performed. Literature searches were conducted in PubMed and Google Scholar. Keyword and MeSH searches included the following: lupus, SLE, systemic lupus erythematosus, dilated cardiomyopathy, and heart failure. Additionally, a hand review of bibliographic references was also performed. Published works between the years of 1990 and 2017 were accepted. Cases in which the patient had previously known SLE or had coronary artery disease leading to heart failure were excluded due to the known prevalence of cardiac involvement in SLE, and the impossibility to exclude an abundance of confounding factors on literature review which could alter interpretation of index of suspicion, diagnosis, and therapeutic response such as degree of cardiovascular disease and prior treatment, prior initiation of immunosuppressive therapy for SLE, the abundance of other clinical manifestations of SLE resulting in end-organ cardiac damage, and lack of measure of cardiac-specific response to therapy.

A total of 11 cases qualifying as SLE debut with heart failure were identified as case reports and case series (Table 2) [10–19]. One case report was excluded due to insufficient information [18]. The average age at diagnosis was 31 years old (range 19–42), and females represented 80% of cases. Common throughout these cases, dyspnea was the most frequent presenting clinical manifestation. Cardiomegaly and markedly decreased left ventricular ejection fractions were described in all cases. However, only four cases were identified in which dilated cardiomyopathy was the only initial manifestation of SLE at the time of presentation, as it was in the aforementioned case.

### 3.1. Differential Diagnosis Considerations

In the context of acute decompensated heart failure, we found that several patients had a protracted course of progressive decompensation, with 3 patients in our retrospective analysis having multiple admissions prior to diagnosis. This echoes findings of the case series performed by Law et al., in which the median time of diagnosis of acute lupus myocarditis from symptom onset was two weeks [20]. Diagnosis was made on serologic basis in all but one case, in which lupus nephritis confirmed on biopsy was the defining factor [15]. This tendency of delayed diagnosis can occur even when connective tissue immunologic profiles are suggestive of an autoimmune etiology to heart failure, as was evidenced in the case series at tertiary care centers described by Tomas et al. [21].

### 3.2. Treatment and Outcomes

All cases reported use of glucocorticoid therapy for immunosuppression, while limited cases added a second immunosuppressive agent, and plasmapheresis was used in one case. All cases in our analysis in which glucocorticoid monotherapy was used resulted in functional recovery, while those adding a second drug or filtration therapy were associated with multiple admissions and a more protracted clinical course in presumably more serious cases. A clinical-functional recovery (CFR), defined as recovery of myocardial function by ECHO or clinical status back to or near previously known functional status, was reported in 90% of cases. The aggressive initiation of immunosuppressive therapy is supported by findings in echocardiography [22] and clinically [20, 21].

## 4. Discussion

The acute presentation of heart failure with DCM in a patient without any of the classic cardiovascular risk factors presents both a medical urgency and a diagnostic challenge. While acute coronary syndrome and pulmonary embolism must initially and quickly be ruled out, a systematic approach provides the building blocks to consider the myriad of possibilities, correctly diagnose, and thereby treat each patient. This includes consideration the less-frequent malignant, autoimmune, and infectious potential causes of disease. This case describing an African-American female whose presentation of SLE was manifested by severe myocarditis and demonstrated a paucity of other clinical findings exemplifies this clinical dilemma.

In the differential considerations of a patient with decompensated heart failure without typical cardiovascular risk factors, we find that, despite several cases being reported, heart failure as the initial manifestation of SLE remains exceedingly rare in the literature, as was observed in a large lupus cohort in Toronto over a period of more than 40 years. Acute lupus myocarditis as the presenting symptom of SLE remains a rare entity, with only 0.37% of patients presenting as such [23]. Due to lack of evidence-based consensus or a comprehensive review of the same, heart failure due to SLE continues to be an underrecognized phenomenon, thereby significantly delaying diagnosis and initiation of treatment.

Within the limited evidence and experience of therapeutic approaches, the efficacy of different singular or combined therapy is also based solely on anecdotal case reports. In compiling and comparing the therapeutic approaches of similar case reports and series in which SLE was found to be the underlying etiology of overt heart failure, we have been able to draw the following conclusions.

Given the near-universal response to a short course of high-dose steroid therapy as much in the clinical recovery as in the described recovery of myocardial function and resolution of DCM, the limited evidence based on review of these observational case studies and series supports the initial use of high-dose steroid therapy in acute lupus myocarditis.

While aggressive immunosuppression is necessary, the poly-pharmaceutical approach is not without risk and the addition of azathioprine and especially cyclophosphamide also adds the potential for significant myelosuppression. The limited evidence based on this literature review of case reports suggests that high-dose steroids offer a reasonable option as a first line of therapy. However, further study with respect to determining the safest and most efficacious approach to acutely heart failure due to lupus dilated cardiomyopathy is required.

## 5. Conclusion

Acute lupus myocarditis is a rare initial manifestation of SLE and an underappreciated cause of acute heart failure due to dilated cardiomyopathy. Limited evidence supports initial and early management with high-dose glucocorticoid therapy, with the majority of patients obtaining clinical and functional recovery. Future investigation with standardized evaluation criteria, treatment regimens, and follow-up is needed to further the management and patient outcomes of dilated cardiomyopathy secondary to lupus.

## Figures and Tables

**Table 1:** Laboratory values of the patient.

Laboratory study	Patient	Reference range
Hemoglobin	9.3^*∗*^	12–16 g/dL
Hematocrit	29.8^*∗*^	36–47%
WBC	6.4	4.0–10 × 10^∧^9/L
Platelets	315	150–450 × 10^∧^9/L
INR	1.4^*∗*^	<1.4
ESR	18	0–20 mm/hr
C-reactive protein	2.9^*∗*^	0–0.8 mg/dL
Creatinine	1.3	0.7–1.3 mg/dL
BUN	42^*∗*^	8–20 mg/dL
ALT	800^*∗*^	0–35 U/L
AST	1074^*∗*^	0–35 U/L
Protein	8.5^*∗*^	6.0–7.8 g/dL
Albumin	3.5	3.5–5.5 g/dL
Troponin	0.237^*∗*^	0–0.1 ng/mL
Pro-BNP	15600^*∗*^	<400 pg/mL
TSH	5.04^*∗*^	0.5–5.0 mU/L
Free T4	1.83	0.9–2.4 ng/dL
Free T3	2.09^*∗*^	3.6–5.6 ng/L
ANA	1 : 1280^*∗*^	<1 : 40
Anti-ds-DNA	7	<30 IU/mL
Anti-Smith	8 AI^*∗*^	Negative
Complement C3	70^*∗*^	88–201 mg/dL
Complement C4	13.1^*∗*^	15–45 mg/dL
Beta-2 glycoprotein	Negative	Negative
Anti-cardiolipin	Negative	Negative
Lyme serology	Negative	Negative
TB quantiferon	Negative	Negative
HIV	Negative	Negative

WBC: white blood cell count; INR: international normalized ratio; ESR: erythrocyte sedimentation rate; BUN: blood urea nitrogen; ALT: alanine aminotransferase; AST: aspartate aminotransferase: pro-BNP: B-type natriuretic peptide; TSH: thyroid-stimulating hormone; ANA: anti-nuclear antibody titer; anti-ds-DNA: anti-double-stranded DNA. ^∗^denotes significant clinical finding.

**Table 2:** Literature review of 9 case reports and one case series reporting heart failure as the presenting sign of systemic lupus erythematosus.

						Diagnostic studies and workup				Treatment					Outcome
Case report	Gender	Age	Pertinent history	Signs & symptoms at presentation	Other manifestations of autoimmune disease	CXR	ECG	Labs	Initial ECHO (EF%)	Steroid	AZA	CYP	Plasmapheresis/IVIG	Clinical	ECHO (EF%)
Ashrafi, 2011	M	35	None	3 months progressive SOB, lower edema swelling	Myositis	DCM	NSR with nonsustained VT	Anti-Ro, anti-La, ANA (1 : 640)	16	Y	N	N	Y	Worsening	Not reported

Baquero, 2011	F	22	FH: CREST	DOE, JVD, lower extremity edema	Lupus nephritis	Bilateral pleural effusions	Sinus tachycardia with nonspecific T wave changes	ESR 71, ANA (1:64), ds-DNA, anti-Smith, low C3, C4	15	Y	N	Y	N	Improved	60%

Sandrasegaran, 1991	F	42	None	Fever x 1 week	None	Congestion, white-out	Mild ST depressions in anterior-lateral leads	Anemia, ESR 150; ANA + (1 : 100); lupus anticoagulant, low C3/C4, anti-Ro	Severe^*∗*^, no EF%	Y	N	N	N	Improved	Reported as improved; no number given

Frustaci, 1996	M	38	None	Fever, palpitations, SOB, weakness x 2 week	Mucosal ulcers	Mod pulmonary congestion	Diffuse ST-T abnormalities	Anemia, ESR 120;	45	Y	N	N	N	None given	65%

Routray, 2004	F	36	None	Dyspnea, orthopnea, tachycardia	None	Pulmonary congestion, left pleural effusion	Diffuse ST-T abnormalities	ESR 80; ANA + (1 : 40); anti-ds-DNA +	35	Y	N	N	N	Improved	45%, ^*∗*^10 day follow-up

Hoang, 2015	F	26	None	Cough, dyspnea, weight loss x 1 month	None	Bilateral lower lobe patchy infiltrates	None	Anemia, AKI, anti-Ro,	35	Y	N	N	N	Improved	60%

Woo, 2009	F	27	None	Dyspnea, orthopnea x 2 weeks	Lupus nephritis	Cardiomegaly, interstitial pulmonary edema	None	Anemia; ESR 33; ANA +; low C3/C4; ds-DNA+, anti-La	30	Y	N	Y, ^*∗*^1 da y	N	Improved	55%

Usalan, 2007	F	32	None	Palpitations, malaise, dyspnea, orthopnea x 10 days	Lupus nephritis, hemolysis	Cardiomegaly, pulmonary congestion bilaterally	Sinus tachycardia with nonspecific ST-T wave changes	Anemia, ESR 90, hemolysis (Coombs direct +), ANA (1 : 160), ds-DNA, low C3/C4, anti-cardiolipin	24	Y	N	N	N	Improved	55%

van der Laan- Baalbergen, 2009	F	19	Arthralgias	Pancytopenia, fever	Proteinuria	Cardiomegaly, pleural effusion	None	Anemia, ANA, ds-DNA, anti-Smith, anti-cardiolipin, low C3/C4	Hypokinesis, pericardial effusion, EF 25%	Y		N	Y	Y	Improved
	F	40	DVT	Dyspnea, alopecia, rash, weakness progressing to cardiogenic shock	Renal Bx: lupus nephritis	Not reported	None	ANA, ds-DNA, Jo, Sm, SSA, SSB, ENA, aCL, cardiac biopsy positive	23	Y	Y	Y	N	Improved	55%

DCM: dilated cardiomyopathy; NSR: normal sinus rhythm; ECHO: transthoracic cardiac ultrasound; EF%: left ventricular ejection fraction; M: male; F: female; Y: yes; N: no; SOB: shortness of breath; DVT: deep vein thrombosis; AZA: azathioprine; CYP: cyclophosphamide; CXR: chest X-ray; Bx: biopsy; DOE: dyspnea on exertion; JVD: jugular vein distention.
